# Dual mode of delivery of *Mycoplasma pneumoniae* CARDS toxin determines the toxin trafficking pathway and cytotoxicity in host cells

**DOI:** 10.1128/mbio.02640-25

**Published:** 2025-11-28

**Authors:** K. Sreenath, Linbo Li, Danielle Molotsky, Jason Ayo, Joel B. Baseman, T. R. Kannan

**Affiliations:** 1 Department of Microbiology, Immunology and Molecular Genetics, The University of Texas at San Antonio 557944, San Antonio, Texas, USA; The University of Alabama at Birmingham, Birmingham, Alabama, USA

**Keywords:** *Mycoplasma pneumoniae*, CARDS toxin, protein secretion, extracellular vesicles, ADP-ribosylation, vacuolation, cytotoxicity, minimal media

## Abstract

*Mycoplasma pneumoniae* (*Mp*) causes both pulmonary and extrapulmonary infections in humans, and its virulence is largely attributed to the community-acquired respiratory distress syndrome (CARDS) toxin. CARDS toxin is transiently expressed during *Mp* growth in the serum-rich SP4 medium, detected predominantly in the cytoplasm, with none secreted into the medium. Here, we show that CARDS toxin synthesis has increased at least fivefold during *Mp* growth in serum-free minimal media, with approximately 40% of the toxin being released into the extracellular environment. In infected human alveolar epithelial cells, the toxin is visualized both in association with *Mp* and in free form around the perinuclear region, confirming its secretion. Using density gradient centrifugation, transmission electron microscopy, and immunoblotting, we demonstrate that *Mp* secretes extracellular vesicles enriched with CARDS toxin. Approximately 35% of the secreted toxin is associated with vesicles, while the remainder exists in a free-soluble form. The vesicle-bound toxin shows increased resistance to proteolytic degradation compared to the free-soluble toxin. Both forms of the purified toxin are biologically active and induce varying degrees of cytotoxicity in human epithelial cells based on their distinct trafficking patterns, which highlights their distinct roles in host-cell interactions. Together, these findings reveal a dual mode of CARDS toxin secretion as a key feature of *Mp* pathogenicity, providing a significant benefit in toxin delivery and thereby enhancing virulence, expanding the scope of infection.

**IMPORTANCE:**

*Mycoplasma pneumoniae* (*Mp*) is a significant human respiratory pathogen whose systemic effects are largely caused by the CARDS toxin. Our study provides new insights into the secretion and delivery of CARDS toxin. We demonstrate that *Mp* highly expresses and selectively releases CARDS toxin when grown in serum-free minimal media and during interaction with host cells. Importantly, *Mp* utilizes two distinct pathways to secrete CARDS toxin: extracellular vesicles (EVs) and a non-EV-mediated route. Compared to the free-soluble toxin, the vesicle-associated CARDS toxin resists proteolytic degradation, which likely enhances its ability to act on distant sites. Both forms of toxin exhibit varying degrees of cytotoxicity on human epithelial cells, highlighting their distinct roles in host-cell interactions. These findings reveal that *Mp* uses multiple strategies to propagate its virulence factors effectively. Understanding these delivery mechanisms provides potential new targets for developing therapeutic interventions to counteract *Mp* pathogenesis, particularly those mediated by CARDS toxin.

## INTRODUCTION

Bacterial toxins are major virulence factors that target host cells and play crucial roles in the host-pathogen dialog (1). To elicit their effects, toxins must be transported across bacterial membranes into the host environment or delivered directly into recipient cells (2). Due to their impact on bacterial colonization and disease progression, secreted toxins are considered promising targets for therapeutic interventions. While extensive research has been conducted on secreted toxins in various bacteria, studies focusing specifically on *Mycoplasma pneumoniae* (*Mp*) remain limited, primarily due to its fastidious growth requirements, low cell yield, and the modest expression of toxin in the complex, serum-rich SP4 media (3).

*Mp* is a small, self-replicating, cell-wall-less, atypical human pathogen with a genome size of 816 kbp, encoding ~677 genes (4, 5). It can cause a range of respiratory infections in children and adults, with clinical manifestations varying from mild illness to severe life-threatening conditions (6). *Mp* accounts for 20% to 30% of all community-acquired pneumonia cases and is also linked to tracheobronchitis, pharyngitis, bronchiolitis, asthma, and chronic obstructive pulmonary disease (6, 7). Infections can result in extrapulmonary manifestations, affecting the neurological, cardiovascular, dermatological, digestive, hematological, and musculoskeletal systems (8). Epidemics of *Mp* infection occur every 3–5 years. A recent epidemiological and transmission modeling study, along with other reports, has highlighted an unprecedented rise in *Mp* detections across multiple countries, particularly in children aged 2–4 years, an age group historically not considered to be at substantial risk for *Mp* pneumonia (9, 10). This shift in the age distribution, coupled with increased hospitalization rates, underscores a concerning post-pandemic trend.

Adhesion is the initial step and prerequisite for *Mp* pathogenicity, enabling attachment to the bronchial ciliary epithelium using both tip- and non-tip-mediated mechanisms (11–13). Key adhesins, such as P1, P30, and P40/P90, along with accessory proteins, facilitate tip-mediated interactions (11, 12, 14, 15), while surface-displayed housekeeping proteins, including elongation factor-Tu (EF-Tu) and pyruvate dehydrogenase B (PDH-B), mediate non-tip-mediated interactions by binding to human fibronectin (13). *Mp* also interacts with human lung surfactant protein A (SP-A), a multifunctional lectin in the bronchoalveolar compartment, through a surface-associated protein MPN372, which was later designated as the community-acquired respiratory distress syndrome toxin (CARDS toxin) due to its central role in pathogenicity (16, 17).

CARDS toxin is a unique, multifunctional toxin that exhibits ADP-ribosyl transferase activity, like diphtheria and pertussis toxins, and vacuolating activity akin to *Helicobacter pylori* VacA (17–19). To elicit these activities, the toxin interacts with host cell surface receptors such as SP-A and/or annexin A2 before entering cells via clathrin-mediated endocytosis (16, 18, 20). By passing through the acidic endosome, it navigates to the endoplasmic reticulum (ER) using the KELED sequence (21, 22). This retrograde transport unleashes the toxin’s distinct N-terminal ADP-ribosylating and C-terminal vacuolating activities that lead to the characteristic cellular damage and inflammation observed in *Mp* infection (21, 22).

Unlike many bacterial toxins, which are encoded within operons, the CARDS toxin in *Mp* is transcribed monocistronically from its promoter (3). CARDS toxin exhibits a temporal gene expression pattern in serum-rich media, with low expression during the early growth stages and a significant decrease as growth progresses (3). In contrast, in experimental animal models, CARDS toxin expression is highly upregulated, driving inflammatory responses and cytopathological effects, suggesting that environmental cues regulate its expression (3, 23). However, the mechanisms underlying toxin expression and its action on target cells remain unclear.

Bacterial pathogens utilize complex protein secretion systems to release virulence factors, including toxins, which enable them to establish colonization, cause acute or chronic infections, subvert the host cell response, and evade the immune system (2). Unlike other bacterial toxins, which are secreted into the extracellular environment via these secretion systems, in SP4 media-grown *Mp* cultures, CARDS toxin is predominantly localized within the cytoplasm, with only a small fraction associated with the cell membrane and no detectable levels in the culture supernatant (3). As a cell-wall-deficient bacterium, mycoplasma is composed of a single biological membrane: protein secretion, therefore, depends solely on the translocation system(s) present within that membrane. *Mp* predominantly utilizes the conserved Sec system for protein secretion, which drives the membrane translocation, insertion, and cleavage of proteins that carry N-terminal signal peptides (4, 5, 24). Furthermore, some *Mycoplasma* species have been shown to secrete extracellular vesicles (EVs) into the external environment, suggesting the existence of alternative protein release pathways (25–27). Although our previous studies have demonstrated the presence of CARDS toxin and *Mp* in the respiratory secretions of infected subjects and animals (3, 23, 28–30) and have investigated the toxin’s host-cell interactions using recombinant toxin (18, 20–22), no studies have yet experimentally reported the active secretion of CARDS toxin or EVs by *Mp*.

In this study, we utilized serum-free minimal media (MM) to propagate *Mp* and elucidate the dynamics of CARDS toxin expression and secretion. For the first time, we demonstrate that CARDS toxin is robustly expressed and selectively secreted during mycoplasma growth in MM, and upon interaction with host cells, highlighting its release as a significant aspect of *Mp* pathogenesis. We also show that *Mp* releases EVs, including those enriched with CARDS toxin, along with free-soluble toxin in the supernatant, implicating a dual pathway of toxin secretion. We further purified both toxin forms to near homogeneity, and they exhibited varying degrees of bioactivity on human epithelial cells, underscoring their distinct roles in host-cell interactions. These findings reshape the understanding of *Mp* pathogenicity and suggest that the dual mode of toxin release may play a crucial role in mycoplasma virulence.

## MATERIALS AND METHODS

### Bacterial cells and mammalian cell culture conditions

*Mp*-S1 strain was routinely grown in 175 cm^2^ tissue flasks containing 80–100 mL of SP4 media under standard conditions (37°C, 95% air, 5% CO_2_) as described before (3). To initiate cultures, a frozen stock from a mid-log phase culture was diluted 1:10 and added to the flasks. Growth curve experiments were performed using either the standard SP4 media or a serum-free minimal medium (MM), which was originally developed by Yus et al. and subsequently optimized by Burgos et al. as described before (31, 32).

For recombinant His-tagged CARDS (rCARDS) toxin expression studies, lipid A-deficient *Escherichia coli* BL21(DE3) (lpxM F^−^ ompT hsdSB [rB^−^ mB^−^] gal dcm) cells harboring the UGA corrected *cards* in pET19b were grown in Luria–Bertani (LB) broth or LB agar containing ampicillin (100 µg/mL) at 37°C as described before (17).

Human cervical adenocarcinoma HeLa cells (CCL-2) and human alveolar adenocarcinoma A549 cells (CCL-185) were purchased from the American Type Culture Collection (ATCC, Manassas, VA, USA) and cultured at 37°C in 95% air-5% CO_2_ in Dulbecco’s modified Eagle medium (DMEM, Thermo Fisher Scientific, Waltham, MA, USA) and F-12K medium (Thermo Fisher Scientific) respectively, supplemented with 10% heat-inactivated fetal bovine serum (FBS, Gibco, Thermo Fisher Scientific), penicillin (100 units/mL), and streptomycin (100 µg/mL) (Life Technologies, Grand Island, NY, USA). All cell lines were maintained in a dedicated tissue culture room to prevent cross-contamination. Cells were routinely screened for mycoplasma contamination using the Venor GeM Mycoplasma Detection kit (Sigma-Aldrich, St. Louis, MO, USA, MP0025-1KT) following the manufacturer’s PCR protocol.

### Growth profile analysis of *Mp* in serum-free media

For comparative analysis of *Mp* growth in SP4 and MM, the S1 strain was initially cultivated in SP4. After about 6 h, when the *Mp* had adhered, the flasks were gently washed with MM, and the cultures were shifted to MM. To assess and quantify the cell-associated and secreted toxins, we collected S1 cells and their corresponding culture supernatants from both SP4 and MM-grown conditions at various time points ranging from 1 to 120 h. For each condition, one flask was set aside for genomic DNA isolation, colony counts, and morphology analysis, while the remaining flasks were used for protein characterization. Metabolic growth analysis and total protein measurements were determined at 24–120 h as previously described (31, 32). For growth analysis, supernatants from triplicate cultures of both SP4 and MM media were collected at every 24 h for 120 h. The metabolic conversion of phenol red was quantified as a growth index, defined as the ratio of absorbance at 430 nm to 560 nm of the culture media. The absorbances were measured using an Accuris Smart Reader 96 microplate absorbance reader (Accuris Instruments, Edison, NJ, USA).

### Membrane fraction purification and analysis

Membrane fractions were isolated from *Mp*-S1 cells grown in SP4 or MM broths using osmotic shock and sonication, as previously described (3). In brief, a 500 mL mid-log phase cultures of *Mp*-S1 cells grown either in SP4 or MM was harvested via centrifugation at 12,000 × *g* for 15 min at 4°C. The resulting cell pellet was resuspended in 1 mL of a hypotonic buffer containing 2 M sucrose. From this suspension, 100 µL was aliquoted for total protein analysis, and the remaining cells (900 µL) were suspended in 250 mL of sterile distilled water to induce osmotic shock. After osmotic lysis, the cell suspension was further homogenized by water sonication (VirSonic 300, Virtis Co INC, Gardiner, NY) for 1 min to ensure complete cell disruption and the release of cellular contents. The resulting cell lysate was separated into membrane and cytosolic fractions by ultracentrifugation at 100,000 × *g* for 1 h at 4°C (Beckman Coulter, Fisher Scientific). The supernatant (cytosolic fraction) was collected, concentrated using an Amicon centrifugal filter (3 kDa molecular weight cutoff; Millipore), and adjusted to 900 µL. The membrane pellet was resuspended and further purified to remove any unbroken cells by using discontinuous sucrose density gradient (30%–60%, wt/vol) centrifugation (Beckman Coulter). After centrifugation, the enriched membrane fraction was collected as indicated before (3, 33), suspended in 900 µL of Tris-NaCl buffer (50 mM Tris-HCl, 150 mM NaCl, pH 7.4), and sonicated for 1 min before being divided into 100 µL aliquots. The membrane yield was calculated relative to the initial total cell lysate using protein estimation (33). To assess the presence of CARDS toxin, cytoplasmic elongation factor-G (EF-G), and membrane adhesin P1, 10 or 20 µL of protein from each fraction was analyzed by immunoblotting, as described in the immunoblot section. The percentage of membrane-associated CARDS toxin was determined from the immunoblot band intensities in each fraction and the membrane yield. The fractions were also analyzed to quantify the percentage of toxin via the toxin capture assay (3). Protein band intensities on immunoblots were quantified by densitometry using ImageJ software (NIH). The average band intensity was calculated from the analysis of three independent immunoblots.

### CARDS toxin secretion and EVs isolation

Culture supernatants collected at 1–120 h were concentrated and analyzed for the presence of CARDS toxin via immunoblot. EVs from the mid-log phase S1 strain grown in MM were isolated via ultracentrifugation as described before (34). Vesicles were further purified by density gradient centrifugation with 10%–45% OptiPrep gradient prepared in 10 mM HEPES and 0.85% NaCl (34). EV-free supernatant was subjected to silver staining to analyze the secreted protein profile, or by antigen capture or immunoblotting for CARDS toxin (3). The pellet was resuspended in PBS and analyzed by transmission electron microscopy (TEM) and by silver staining and immunoblotting, to examine the presence of EVs, protein profile, and CARDS toxin, respectively. Purified fractions were subjected to TEM to analyze the EVs, or dot blot to examine CARDS toxin. The CARDS toxin concentration in these collected fractions was determined using an antigen capture assay as previously described (3, 23).

### Purification of native free-soluble and recombinant CARDS toxin

The secreted CARDS toxin from the EV-free soluble supernatants was purified by ammonium sulfate precipitation, anion exchange, and size-exclusion chromatography (35, 36). rCARDS toxin was purified using Ni-NTA (nickel-nitrilotriacetic acid) column chromatography (17, 22).

### Total protein measurement

Total protein concentrations were determined using a Bradford assay kit (Thermo Fisher Scientific). The assay was performed on total cell lysates, membrane fractions, and cytosolic fractions isolated from SP4 and MM cultures. Additionally, protein concentrations were measured for the supernatant and EV fractions collected from the MM culture.

### Trypsin treatment of free and vesicle-associated CARDS toxin

Limited protease digestion of CARDS toxin associated with EVs, and the vesicle-free supernatant was performed using trypsin, as described previously (18).

### Immunoblot analysis

All protein samples were resolved by 12% SDS-PAGE or 4%–12% Nu-PAGE gradient gels and stained with Coomassie brilliant blue or silver stain (Invitrogen) for visualization. A parallel gel of separated proteins from each experimental sample was transferred onto a 0.2 µm nitrocellulose membrane (Bio-Rad Laboratories, Hercules, CA, USA) using semi-dry transfer (Bio-Rad Laboratories) for immunoblotting. After blocking in 5% milk, membranes were probed for the presence of CARDS toxin (anti-rabbit antisera 1:7,000), cytoplasmic EF-G (anti-rabbit antisera 1:1,000, gift from Dr. Richard Herrmann), membrane adhesin P1 (anti-mouse monoclonal antibody 1:200), and cytosolic EF-Tu (anti-rabbit antisera 1:1,000) overnight at 4°C. Membranes were then treated with alkaline phosphatase (AP)-conjugated or peroxidase-conjugated secondary goat anti-rabbit antibody, or goat anti-mouse antibodies (1:5,000; Invitrogen) for 1 h and visualized by colorimetric or chemiluminescent analysis using an ECL imaging system (Bio-Rad ChemiDoc MP), respectively.

### Immunofluorescence microscopy and vacuolation analysis

To study the expression and localization of CARDS toxin during *Mp* infection, A549 alveolar epithelial cells were infected with mCherry *Mp*-S1 stocks. To create a uniform inoculum, the mycoplasmas were passed five times through a 25-gauge needle and then sequentially filtered through 0.8 µm and 0.4 µm filters. Before infection, stocks were quantified by dilution and plating.

For the infection experiment, A549 cells were seeded at a density of ~1 × 10^5^ cells/well, and at 60%–70% confluency, *Mp* was added to each culture well at a multiplicity of infection (MOI) of 40:1 (40 *Mp* per A549 cell) and incubated for 4 h before fixation.

IFA was performed as described previously (21). Briefly, infected and control A549 cells were fixed with 4% paraformaldehyde, permeabilized with 0.1% Triton X-100, and then blocked with 1% normal goat serum (Gibco). To detect CARDS toxin, cells were incubated with rabbit polyclonal anti-CARDS toxin antibodies (1:2,000) followed by Alexa Fluor 488 (AF 488)-conjugated goat polyclonal anti-rabbit secondary antibodies (1:500; Invitrogen). Nuclei were stained using DAPI (Vector Laboratories), and *Mp* was detected using mCherry channel. Optical sections of DAPI, AF 488, and mCherry were imaged using a multichannel acquisition system operated by AxioVision on a Carl Zeiss Z.1 Cell Observer microscope.

In contrast to A549 cells, HeLa cells exhibit minimal basal vacuole formation. To accurately measure the effects of low toxin concentrations, we, therefore, chose HeLa cells for both IFA and vacuole formation studies. This approach allows for a clear correlation between toxin transport and vacuole development. For the toxin IFA studies, HeLa cells were seeded at a density of approximately 1 × 10^5^ cells per well onto coverslips in 12-well plates and grown to 70%–80% confluency. To study the localization of free-soluble or EV-associated CARDS toxin, cells were treated with each sample containing 70 pmol of CARDS toxin (5 µg of CARDS/mL) for 6 h. rCARDS toxin (70 pmol; 5 µg/mL) was used as a positive control. After intoxication, cells were fixed and permeabilized and blocked as described above. CARDS toxin distribution was analyzed using indirect immunofluorescence with anti-CARDS toxin antibodies (1:2,000) and an AF 555-conjugated secondary antibody (1:500) (Invitrogen). F-actin was stained with AF 488-labeled phalloidin (Invitrogen). For co-immunofluorescence analysis (co-IFA), cells were labeled with monoclonal antibodies against GM130 (1:1,000, Abcam ab1299), as previously described (21, 22). Samples were incubated with an AF 488-conjugated secondary antibody and mounted in a medium containing DAPI. Images were captured as previously reported using AxioVision software and enhanced in Adobe Photoshop.

To investigate cytotoxicity induced by secreted CARDS toxin, HeLa cells were grown to 60%–70% confluency in six-well plates (seeded at ~3 × 10^5^ cells/well) or T25 flasks (seeded at ~1 × 10^6^ cells). They were then treated for 24 h with either EV-free native toxin, recombinant toxin, or a purified CARDS toxin-enriched EV-fraction (each containing 2.5 or 5 µg of toxin/mL; 35 and 70 pmol). Carrier buffer served as the negative control. Vacuole formation was then analyzed manually in triplicate experiments by assessing the vacuolization patterns, including the timing, size, and the number of vacuolated cells, across 10 fields with 20–25 cells per sample as previously described (22, 37).

### Statistical analysis and reproducibility

All experiments were performed at least three times, and representative data were shown. Results are presented as the mean ± standard error of the mean of triplicates. We compared multiple groups using a one-way analysis of variance, accompanied by Tukey’s multiple-comparison post hoc test. Comparison between two groups was analyzed by an unpaired two-tailed *t*-test. All statistical analyses were conducted using GraphPad Prism v10 (GraphPad Software, San Diego, CA), with *P* < 0.05 considered significant.

Detailed experimental protocols have been provided in the Supplemental Materials and Methods section.

## RESULTS

### Optimization of *Mp* growth under serum-free conditions and CARDS toxin synthesis

**Fig 1:**
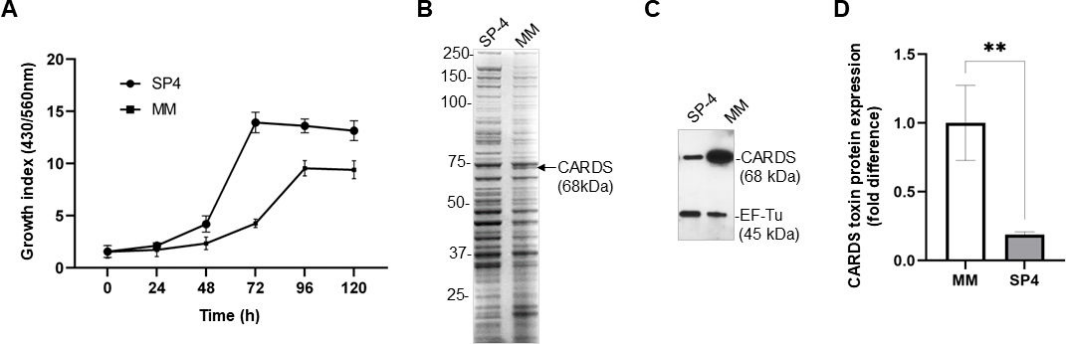
Effect of media composition on *Mp* growth and CARDS toxin expression. (**A**) Growth of *Mp* in SP4 vs MM broth. Metabolic growth index was measured at specific time intervals (24–120 h) using the ratio of absorbance at 430 nm to 560 nm. (**B**) Comparison of total protein profiles of *Mp* grown in SP4 vs MM. Total cell lysates (20 µg) from *Mp*-S1 cells were separated using SDS-PAGE gel and stained with Coomassie brilliant blue. The arrow indicates a prominent 68 kDa protein detected in MM-grown cell lysates. (**C**) CARDS toxin expression. A gel ran in parallel to that in panel B was used for immunoblotting and probed with antibodies against CARDS toxin and EF-Tu. EF-Tu served as a loading control. (**D**) Quantification of CARDS toxin protein. The fold differences in CARDS toxin protein levels between *Mp* grown in MM and SP-4 broth at mid-log phase, normalized to EF-Tu levels. **P < 0.006.

The propagation of *Mp* in serum-rich SP4 media poses challenges with the purification and characterization of secreted proteins; therefore, we used serum-free minimal medium (MM) as a potential alternative. In SP4 medium, mycoplasma reached the stationary phase at 96 h, whereas in MM, it extended to 120 h (Fig. 1A) while maintaining ~67% of the total biomass compared to SP4. The overall protein profiles in total cell lysates were largely similar between the two media, although a noticeable variation was observed in the abundance of a few proteins, including a 68 kDa protein overexpressed in MM (Fig. 1B). This 68 kDa protein was identified as CARDS toxin by parallel immunoblot analysis, which confirmed the toxin overexpression in MM compared to SP4 (Fig. 1C). The expression of EF-Tu, served as a loading control, remained similar across both media conditions, indicating that the observed increase in CARDS toxin expression was specific to the serum-free media. Densitometric quantification estimated a ~5-fold increase (*P* < 0.006) in CARDS toxin levels in MM compared to SP4 (Fig. 1D). Furthermore, the toxin capture assay of total protein cell lysates revealed a nearly 4.5-fold increase in toxin yield in MM (2.37 ± 0.68 ng/µg of lysate) compared to SP4 media (0.53 ± 0.15 ng/µg of lysate). Overall, the data indicate that the serum-free MM effectively supports large-scale cultivation of *Mp*, providing a reliable platform for producing sufficient *Mp* proteins, especially for the efficient production of CARDS toxin, for downstream analyses.

### CARDS toxin localization in *Mp* under serum-free growth conditions

**Fig 2:**
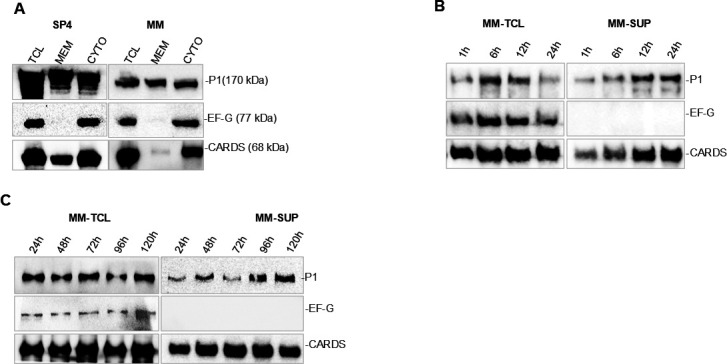
Localization and secretion of CARDS toxin in *Mp* grown in minimal media (**A**) Cell fractionation of *Mp* and localization of CARDS toxin. *Mp* was grown in either MM or SP4 medium. Total cell lysate (TCL), membrane (MEM), and cytoplasmic (CYTO) fractions were isolated, and proteins from each fraction were separated by SDS-PAGE and transferred to nitrocellulose membranes. Immunoblotting was performed to detect the presence of CARDS toxin, adhesin P1 (membrane marker), and EF-G (cytoplasmic marker). (**B and C**) Expression and secretion of CARDS toxin. *Mp* was grown in MM. Total cell lysates (TCL) and culture supernatants (SUP) collected at specific points (1 h−24 h [**B**] and 24 h–120 h [**C**]) were subjected to immunoblotting to detect CARDS toxin, P1, and EF-G.

To examine the subcellular localization of CARDS toxin, total cell lysates, membrane, and cytosolic fractions of mid-log phase cultures of SP4- and MM-grown *Mp* cells were isolated and subjected to immunoblot analysis. In MM-grown *Mp*, only ~2%–3% of CARDS toxin was detected in the membrane fraction, whereas in SP4-grown cells, ~10%–14% of the toxin was associated with the membrane (Fig. 2A). These results indicate that under MM growth conditions, CARDS toxin is predominantly localized in the cytosolic fraction, and only a small percentage is associated with the membrane.

### Verification of secreted CARDS toxin

Although CARDS toxin is highly expressed in MM-grown *Mp*, its decreased association with the mycoplasma membrane fraction prompted us to investigate its release in the supernatant. As shown in Fig. 2B, immunoblotting using an anti-CARDS toxin antibody detected a single strong band at ~68 kDa in the concentrated supernatant, consistent with the protein size detected in the whole-cell lysate, indicating that the full-length toxin is released. P1 was also detected in the supernatant along with CARDS toxin at all analyzed time points (Fig. 2B and C). However, EF-G, the cytosol-associated protein, was not found in the supernatant, indicating minimal cell lysis during the analyzed time frames (Fig. 2B and C). Despite its release, abundant levels of CARDS toxin were detected along with P1 and EF-G in the total cell lysate throughout the growth phases (Fig. 2B and C). When compared to cell-associated CARDS toxin, almost 40% of the toxin was detected in the supernatant. These findings indicate that a significant amount of CARDS toxin is secreted during *Mp* growth in serum-free media, rather than being released through autolysis.

### Secretion of CARDS toxin during *Mp* host cell interaction

**Fig 3:**
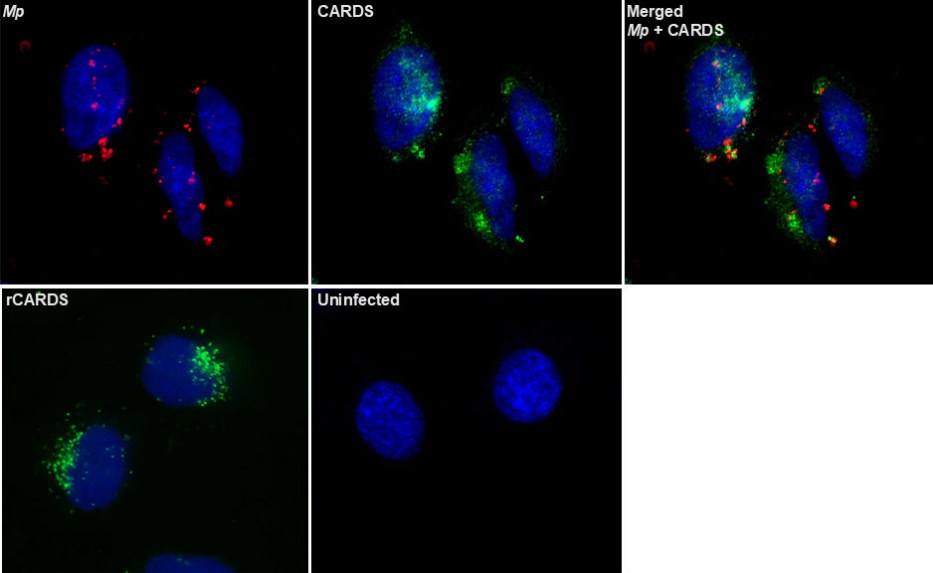
Synthesis and distribution of CARDS toxin in *Mp*-infected A549 alveolar epithelial cells. A549 cells were infected with mCherry-expressing *Mp* at an MOI of 40:1. The distribution of *Mp* and CARDS toxin was analyzed by IFA at 4 h post-infection. As a positive control for toxin localization, a separate batch of A549 cells was treated with 140 pmol of recombinant CARDS toxin (rCARDS). *Mp* was visualized via mCherry fluorescence (red). CARDS toxin was detected using a rabbit polyclonal anti-CARDS toxin antibody, followed by an AF 488-coupled secondary antibody (green). Cell nuclei were counterstained with DAPI (blue). *Mp* uninfected cells probed with CARDS toxin antibody served as a negative control.

Although our previous study demonstrated the upregulation of CARDS toxin transcripts in infected epithelial cells, it did not directly analyze the secretion of CARDS toxin (3). Therefore, we examined its synthesis, secretion, and distribution during the alveolar epithelial cell interaction of *Mp* using IFA as described in the Materials and Methods. At 4 h post-infection (PI), the mCherry *Mp* was readily detected on the surface of infected epithelial cells (Fig. 3). Parallel IFA for CARDS toxin revealed the toxin production (Fig. 3), and the mCherry-*Mp* merged images showed not only the mycoplasma-associated CARDS toxin but also the dissemination of the toxin in areas distinct from the bacteria, suggesting its release during *Mp*-host cell interaction (Fig. 3). Intriguingly, regardless of the low MOI (40:1) and the distribution of bacteria, abundant mycoplasma*-*free CARDS toxin was readily detected around the perinuclear region of infected cells, demonstrating its high expression during infection and a trafficking pattern consistent with that of purified rCARDS toxin (Fig. 3).

### Association of CARDS toxin with *Mp* EVs and EV-free supernatant

**Fig 4:**
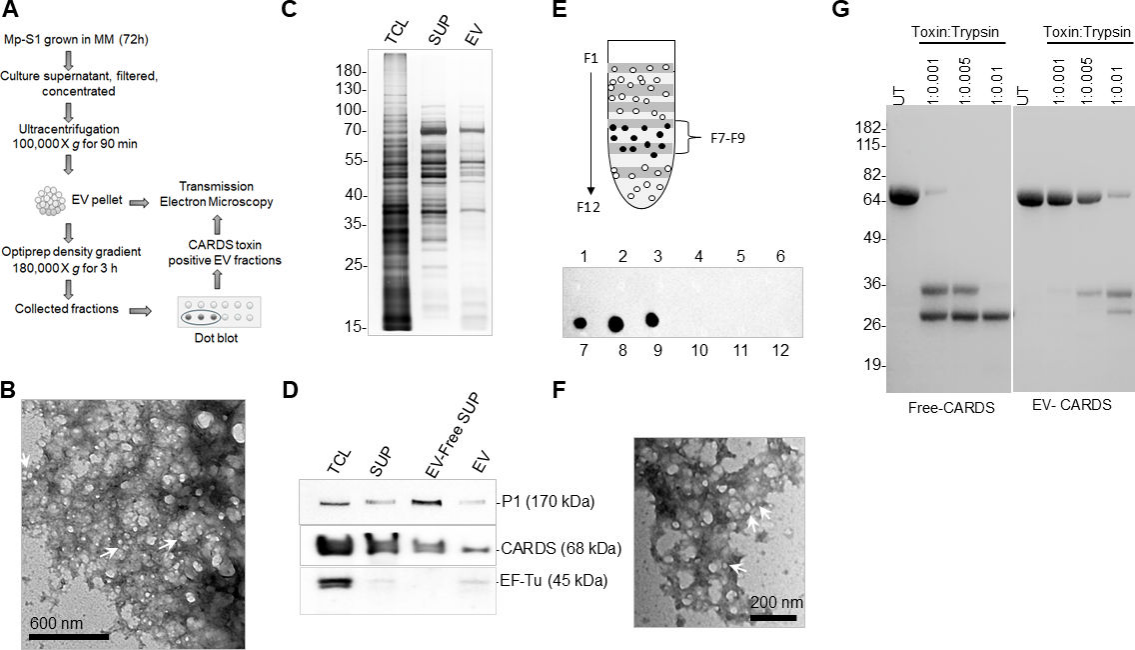
Analysis of CARDS toxin in *Mp*-derived vesicles and vesicle-free supernatants. (**A**) Schematic of EV isolation and purification. (**B**) Transmission electron microscopy (TEM) analysis of EV pellet. The spherical, intact vesicles are indicated by arrows (Scale bar = 600 nm). (**C**) Analysis of secreted protein profiles of *Mp*. Total cell lysate (TCL), supernatant (SUP), and EVs isolated from MM-grown *Mp* were resolved on a 12% SDS-PAGE and silver-stained for visualization. (**D**) A parallel gel transferred to a nitrocellulose membrane was examined by immunoblotting for the presence of CARDS toxin, P1, and EF-Tu. (**E**) Density gradient purification of CARDS toxin-containing EVs. EV pellet (from B) was subjected to OptiPrep density gradient to separate EVs based on size and density. The sequentially collected fractions were then analyzed by immunoblotting for the presence of CARDS toxin. (**F**) TEM analysis of purified EVs (CARDS toxin-containing EV fractions). Arrows indicate intact, spherical vesicles with a size range of 30–70 nm (Scale bar = 200 nm). (**G**) Limited trypsin digestion of EVs and EV-free supernatant followed by immunoblot analysis of CARDS toxin. The ratio of trypsin to EV or free soluble CARDS toxin is indicated above each lane.

As virulence factors that do not contain an export signal can be secreted through membrane-blebbed vesicle-mediated transport, we investigated whether *Mp* can form EVs enriched with CARDS toxin. To analyze the presence of EVs, mid-log phase MM-grown *Mp* culture supernatants were subjected to EV purification as described in the Materials and Methods and illustrated in Fig. 4A. Transmission electron microscopy (TEM) analysis of the collected initial pellet (Fig. 4A) showed spherical-shaped vesicles of varying sizes (30–200 nm), indicating that *Mp* naturally produces EVs under serum-free conditions (Fig. 4B). Silver stain analysis of total cell proteins, EVs, and EV-free soluble secreted protein fractions revealed distinct protein profiles implicating the selective nature of protein packaging in EVs (Fig. 4C). Parallel immunoblot analysis detected CARDS toxin in all protein fractions, indicating that the toxin exists not only as a soluble secreted protein but also associated with EVs (Fig. 4D). The P1 adhesin and EF-Tu were also detected in the EV fraction (Fig. 4D). Densitometry analysis determined that the EVs contained ~35% of the total secreted CARDS toxin (Fig. 4D). Furthermore, separation of crude EVs using a stepwise Optiprep gradient ultracentrifugation, followed by dot blot analysis, readily demonstrated the presence of CARDS toxin only in a specific subset of the collected fractions (Fractions 7–9; Fig. 4E). Subsequent TEM examination of these fractions confirmed the presence of vesicles with a characteristic morphology, consisting of spherical nanostructures surrounded by a membrane, with a diameter ranging from 30 to 70 nm (Fig. 4F).

### Differential susceptibility of EV-free and EV-associated CARDS toxin to protease digestion

As previous studies with other bacteria have shown that EVs function as protective transport vesicles for toxin delivery (38, 39), we investigated whether EV association confers protection against protease cleavage for CARDS toxin. To test this, we treated EV-associated and free-soluble CARDS toxin with trypsin. As seen in the immunoblot, limited trypsin digestion cleaved the free soluble CARDS toxin into smaller fragments [~35 kDa and ~33 kDa, corresponding to N- and C-terminus regions of CARDS (18)] in a concentration-dependent manner, as shown in Fig. 4G (left panel). In contrast, the EV-associated CARDS toxin was relatively stable at lower concentrations of trypsin (Fig. 4G, right panel). At higher doses, the toxin was cleaved into the fragments as mentioned earlier, suggesting that the EV-encapsulation protects most of the toxin from proteolytic degradation.

### Purification of EV-free CARDS toxin

**Fig 5:**
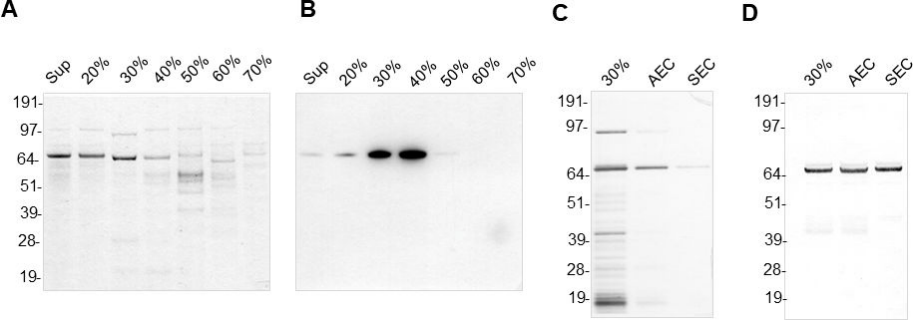
Purification of vesicle-free CARDS toxin. Effect of ammonium sulfate concentration on the precipitation of free-soluble CARDS toxin. EV-free supernatant, collected from MM-grown *Mp*-S1 cells at mid-log phase, was subjected to ammonium sulfate precipitation. Proteins precipitated at 20%–70% ammonium sulfate saturation were resolved on a 4%–12% Nu-PAGE gradient gel and analyzed by (**A**) Coomassie silver staining or (**B**) immunoblotted using anti-CARDS toxin antibodies. Lanes correspond to the vesicle-free supernatant (Sup) and the ammonium sulfate concentration used for protein precipitation (20%–70%). (**C**) CARDS toxin-enriched fraction (30% & 40% ammonium sulfate saturation) was subjected to anion exchange chromatography (AEC) followed by size exclusion chromatography (SEC). Purified CARDS toxin from each step was concentrated, resolved on a 4%–12% Nu-PAGE gradient gel, and analyzed by silver staining. (**D**) A parallel gel of separated proteins (Panel **C**) was transferred to a nitrocellulose membrane and immunoblotted for CARDS toxin using anti-CARDS toxin antibody, followed by secondary antibody conjugated with AP.

Due to their inherent slow growth, minimal cell yields, and complex serum-rich media, attempts were not made to purify secreted proteins under their native conditions from *Mp*. Having detected abundant levels of free-soluble CARDS toxin (~65% of the total secreted toxin), in the *Mp*-grown MM culture supernatant, we employed a combined approach of ammonium sulfate precipitation, anion exchange chromatography, and gel filtration to purify the free-soluble toxin from a large-scale culture as described before for diphtheria toxin (35). CARDS toxin was selectively enriched in fractions containing 30% and 40% ammonium sulfate (Fig. 5A and B). The enriched toxin fractions were combined and further purified by using an anion exchange and size exclusion chromatography columns. The eluted fractions on the silver-stained gel revealed the stepwise purity of the toxin (Fig. 5C). Immunoblot analysis further confirmed the homogeneity of the purified free-soluble toxin (Fig. 5D).

### Host cell interaction of EV-free and EV-associated CARDS toxin

**Fig 6:**
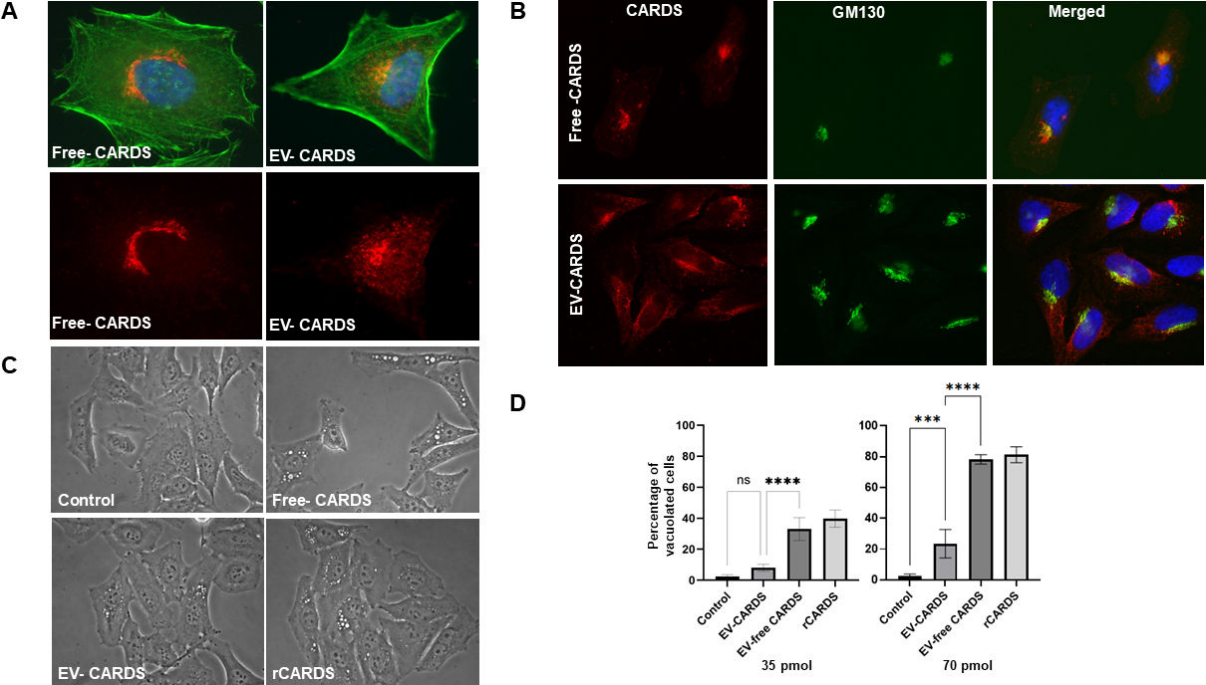
Interaction of vesicle-associated and free soluble CARDS toxin with host epithelial cells. (**A**) Distribution of EV-associated and EV-free CARDS toxin. HeLa cells were treated with 70 pmol of either EV-associated or EV-free CARDS toxin for 6 h. CARDS toxin was stained with AF 555 (red), F-actin with AF 488-conjugated phalloidin (green), and nuclei with DAPI (blue). (**B**) Golgi apparatus colocalization of EV-associated and EV-free CARDS toxin. HeLa cells were treated with EV-associated or EV-free CARDS toxin for 6 h and stained for CARDS toxin (red) and nuclei (blue) as described above. The Golgi apparatus was probed with a mouse monoclonal anti-GM130 antibody, followed by an AF 488-conjugated secondary antibody (green). (**C**) EV-associated and vesicle-free CARDS toxin-mediated vacuolation in HeLa cells. HeLa cells were treated with 35 and 70 pmol of EV-associated or EV-free or rCARDS toxin, along with carrier buffer (served as control) for 24 h. Representative images of vacuolated cells generated by CARDS toxin at 24 h are shown. (**D**) Quantification of CARDS toxin-induced vacuolated HeLa cells. As described in Materials and Methods section, vacuolated cells were counted and expressed as a percentage of control cells. ns, not significant; ***P < 0.0003 and ****P < 0.0001.

As purified rCARDS toxin induces cytotoxicity, including vacuolating activity, by passing through the retrograde pathway (21, 22), we aimed to determine if both EV-free and EV-associated CARDS toxins are biologically active. In an initial experiment, HeLa cells were treated with purified free-soluble CARDS toxin or EV-associated CARDS toxin, and their trafficking patterns were evaluated by IFA. As shown in the IFA, EV-associated CARDS toxin was detected as individual puncta throughout the cells, with a moderate accumulation around the perinuclear region. In contrast, most of the soluble CARDS toxin was detected around the perinuclear region at 6 h (Fig. 6A). Organelle co-localization studies revealed that almost all free-soluble CARDS toxin co-localized with the Golgi apparatus, whereas only a small percentage of vesicle-associated CARDS toxin co-localized with the Golgi apparatus by 6 h (Fig. 6B).

To further assess the impact of toxin transport on the cytotoxicity of secreted CARDS toxins on target cells, HeLa cells were treated with two different dosages of purified free-soluble and EV-associated CARDS toxins, and the vacuolating activity was estimated as indicated in the Materials and Methods. At the low dose (35 pmol), the EV-associated toxin induced a low level of vacuolation (~8%) compared to carrier solution-treated control cells (Fig. 6D). In contrast, the free-soluble toxin induced significantly higher vacuolation (~32%, *P* < 0.0001), like the recombinant toxin (~40%, *P* < 0.0001) (Fig. 6D). At the high dose (70 pmol), a considerable increase in vacuolation was observed across all treatments. Compared to the control, the EV-associated CARDS toxin induced vacuolation in approximately 25% of the cells (*P* < 0.003). However, the free-soluble toxin induced significantly more vacuoles in cells (≥80%), similar to rCARDS toxin (≥85%), and this effect was statistically higher than the EV-associated toxin (*P* < 0.0001). Vacuolation was stably maintained throughout all analyzed time intervals (Fig. 6C and D). Overall, the variations in EV and soluble CARDS toxin uptake influence the toxin transport pathway and modulate the vacuole formation.

## DISCUSSION

This study provides new insights into the expression and secretion of CARDS toxin from *Mp*. For the first time, we show the robust expression and selective release of CARDS toxin under serum-free conditions. CARDS toxin secretion is significantly enhanced during *Mp* infection of alveolar epithelial cells, highlighting its role in host-pathogen interactions. We also uncovered a dual secretion pathway for CARDS toxin, with about ~35% of the secreted toxin bound to EVs, while the rest exists in a free, soluble form. Furthermore, the purification and functional characterization of both toxin forms show their ability to engage host cells and induce varying levels of cytotoxic effects, underscoring their pathogenic potential. This work also highlights the first successful purification of a secreted toxin from *Mp,* offering potential for future diagnostic and therapeutic applications.

### Enhanced expression and synthesis of CARDS toxin

Environmental factors, such as temperature, pH, osmolarity, and nutrient availability, can modulate the expression of virulent genes in bacterial pathogens. Our previous work in serum-rich media has shown that CARDS toxin expression peaks transiently during the early logarithmic phase of *Mp* growth and diminishes thereafter (3). Here, we show that under serum-free conditions, CARDS toxin synthesis is constitutive until the late logarithmic phase, resulting in more sustained and robust expression. This adaptive response to nutrient-deprived conditions (like serum-free medium) is observed in other bacterial pathogens as well, where specific environmental conditions enhance the production of virulent proteins. For example, culturing *Staphylococcus aureus* in a chemically defined medium leads to the upregulation of hemolysins, enterotoxins, and proteases (40). Similarly, *Pseudomonas aeruginosa* grown in a minimal medium with limited inorganic phosphate exhibits increased accumulation of virulence proteins related to pyocyanin synthesis, secretion systems, quorum sensing, and chemosensory signaling (41). A defined medium has also been described for enhancing exotoxin A production in *P. aeruginosa*, with divalent cations and glycerol influencing the toxin yield (42). Our study suggests that specific environmental conditions, like nutrient limitation, might trigger a stress response in *Mp*, prompting the active synthesis of CARDS toxin in MM. However, the absence of this effect during the stationary phase of SP4 cultures (3), when nutrients are also depleted, indicates that the regulatory mechanism is more complex than simple nutrient exhaustion and is likely tied to specific media components. Further study exploring the precise nutritional and environmental factors within MM that contribute to this increased toxin yield could lead to a deeper understanding of *Mp* pathogenicity and potential strategies for controlling toxin production in the context of infections.

Gene expression regulation in *Mp* remains complex, particularly in how it coordinates the synthesis of essential proteins in response to environmental changes. While our previous study demonstrated that *Mp* employs the repressor HrcA and CIRCE (controlling inverted repeat of chaperone expression) to regulate the expression of heat shock proteins like ClpB (43), the regulatory mechanisms governing CARDS toxin expression remain less understood. However, CARDS toxin expression is controlled by environmental cues and is significantly upregulated during infection compared to growth in SP4 medium (3, 23). Studies that investigated transcription regulation in *Mp* identified several key transcription factors (TFs), and subsequent studies revealed their targets using an array of *Mp* mutant strains under various genetic and environmental conditions (44–47). However, the specific TF(s) and regulatory protein(s) that modulate CARDS toxin expression are still unknown. Further research is needed to unravel the intricacies of CARDS toxin regulation, including the identification of the regulatory elements in this genome-reduced pathogen. Given the robust expression of CARDS toxin in MM, this system offers an ideal platform for elucidating the regulatory mechanisms governing toxin biosynthesis.

### Secretion of CARDS toxin

**Fig 7:**
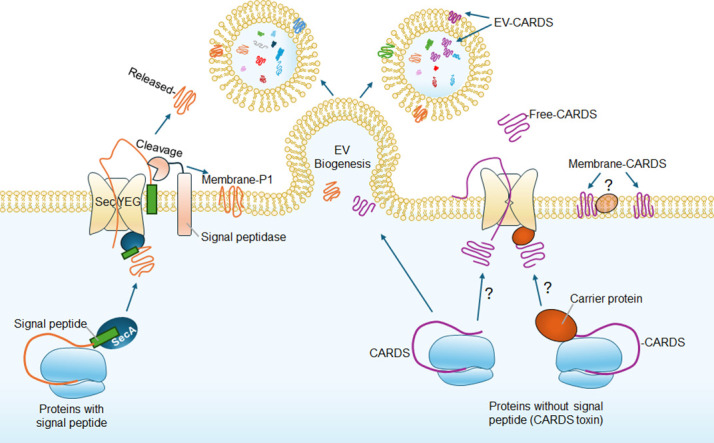
Secretion of *Mp* CARDS toxin mediated by vesicle-associated and vesicle-free pathways. Proposed model for *Mp* EV biogenesis and CARDS toxin secretion. *Mp* secretome consists of both exoproteins and the EV cargo. *Mp* possesses the genes for the Sec-pathway-dependent export. Exoproteins with signal peptides, such as P1, are likely processed by the Sec machinery and translocate through SecYEG into the membrane or are released to the extracellular environment after cleavage of their signal peptide by signal peptidases. Exoproteins lacking signal peptides, like CARDS toxin, are likely transported by carrier proteins and translocate into the membrane or are released via the Sec machinery or an unknown pathway. EVs are formed from the single plasma membrane of this cell wall-less bacterium, with biogenesis likely modulated by environmental factors, such as nutrient availability. During EV biogenesis, specific cytoplasmic proteins, membrane-associated proteins, and exoproteins are selectively packaged into the EVs (either within the lumen or associated with the EV membrane). These EV cargos exist with or without CARDS toxin.

Both Gram-positive and Gram-negative bacteria rely on a variety of secretion systems to deliver effector proteins into host cells, whereas the cell wall-deficient *Mp* relies predominantly on the Sec system for protein secretion, with genome reannotation confirming the presence of nearly all Sec complex components (5, 48) (Fig. 7). In most cases, *Mp*-encoded proteins that contain an N-terminal signal peptide recognized by the Sec system, including the adhesin protein P1, are efficiently anchored to the cell membrane or secreted (48, 49). Intriguingly, despite the lack of a signal peptide, CARDS toxin is readily detected on the membrane or secreted into the extracellular environment (Fig. 2B, C, and 7). Mounting evidence suggests that pathogens such as *Mycobacterium tuberculosis* employ alternative SecA2-dependent mechanisms to secrete proteins lacking signal peptides, including superoxide dismutase (50). Similarly, *Listeria monocytogenes* secretes proteins without signal peptides (e.g., DnaK, GroEL, pyruvate dehydrogenase E2 subunit, enolase, EF-Tu) via the SecA2-dependent pathway (51). These findings support the notion that *Mp* may utilize a similar or yet unidentified pathway for the secretion of proteins, such as the CARDS toxin. Hence, identifying the element(s) that promote CARDS toxin secretion would shed light on the alternative secretion mechanism of *Mp*.

### Dual modes of CARDS toxin secretion

To compensate for the lack of other secretion systems, *Mp* sheds EVs, which may serve as an alternative secretory pathway in delivering virulence factors (Fig. 7). EVs play a critical role in delivering bacterial toxins in many pathogens, including heat-labile toxin from Enterotoxigenic *E. coli*, pneumolysin from *Streptococcus pneumoniae*, and cholera toxin from *Vibrio cholerae* (39, 52, 53). Our findings indicate that *Mp* also employs a similar strategy to deliver not only the CARDS toxin but also the P1 adhesin and the fibronectin-binding EF-Tu via vesicles. Strikingly, the presence of CARDS toxin in specific vesicle fractions, as observed in gradient separation, underscores the molecular complexity and heterogeneity of the cargo in these vesicles. This selective packaging may play a critical role in enhancing the ability of *Mp* to establish and maintain infection by facilitating the efficient delivery of the toxin to target host cells. Similar selective cargo packaging in EVs has been reported in other bacterial pathogens (54, 55). Further characterization of these purified vesicle cargoes and investigating their individual and combined effects on host cells may provide valuable insights into *Mp* survival, virulence, and dissemination.

Of the total secreted CARDS toxin, around ~35% is associated with vesicles, while the rest exists as a free-soluble form. Similar dual-release mechanisms have been observed for other bacterial toxins, such as cholera toxin from *V. cholerae,* listeriolysin O from *L. monocytogenes*, VacA from *H. pylori*, cytolysin A from *E. coli*, and cytolethal distending toxin from *Campylobacter jejuni*, where the EV-associated toxin ranges between ~10%–30% (39, 56–58). The presence of both vesicle-associated and free-soluble forms suggests that *Mp* utilizes multiple mechanisms for releasing toxins and interacting with host cells.

In *Mp*, most of the toxin was detected within the cytosol, with a small fraction found on the surface, implicating its distribution primarily within the vesicle lumen during EV biogenesis. Compared to the free toxin, vesicle-associated CARDS toxin showed increased resistance to protease degradation, consistent with observations in other pathogens, where vesicle encapsulation protects toxins from host proteases (39). In the airway tract, free CARDS toxins may be readily cleaved by trypsin, chymotrypsin, and neutrophil elastases. In contrast, the vesicle-enclosed toxin may be better protected from these host proteases, likely enhancing its stability and efficacy during infections. This aligns with the detection of CARDS toxin in respiratory samples from *Mp*-infected individuals, even at low infection doses (29), suggesting that vesicle-mediated encapsulation might enhance toxin’s persistence and *in vivo* activity. CARDS-toxin-containing vesicles may enter circulation from the primary site of infection, enabling dissemination to distant target organs and resulting in possible extrapulmonary manifestations, which are reported in ~25% of *Mp*-infected individuals.

### Vesicle-associated and -free toxin interact with host cells

In epithelial cells, EV-associated CARDS toxin binds, internalizes, and accumulates intracellularly, similar to its EV-free counterpart. While the toxin is primarily enclosed within vesicles, evidence from membrane fractionation experiments (Fig. 2A) and our previous studies (3, 16) also indicates its localization on the surface of the *Mp* membrane. This membrane distribution suggests that a small fraction of the toxin may also be localized on the surface of the EVs. This surface-exposed toxin could function as both a virulence factor and an adhesin, potentially interacting with host cells alongside with other vesicle-associated adhesins like P1 and EF-Tu. Future studies are needed to further characterize these vesicle-associated virulence proteins, determine their precise localization within the EVs, and investigate their impact on host-cell interactions and pathogenesis.

Free-soluble toxin elicited more vacuoles than its vesicle-associated counterpart. This difference is likely due to trafficking dynamics. Free-soluble toxin interacts with specific host cell surface receptors (16, 19, 37), which facilitate its entry and direct it through the retrograde trafficking pathway (21). This pathway, along with an acidic environment, is crucial for the clipping of the toxin, which is required for the downstream ADP-ribosylating and vacuolating activities (22). In contrast, EV-associated toxins might utilize different entry mechanisms, depending on factors like the size of EV, surface cargo proteins, and interacting host cell type. This could result in diverse trafficking patterns and reduced access to the specific retrograde pathway needed for efficient activation. Therefore, the differential trafficking patterns of free-soluble vs vesicle-associated CARDS toxin, specifically regarding their retrograde transport and subsequent processing, appear to be a key factor in explaining the observed difference in vacuolating activity. While this explains the observed difference, the possibility that vesicle association confers unique functionalities upon the toxin must also be investigated in future studies.

In conclusion, our study shows that CARDS toxin is highly expressed and selectively secreted by *Mp* under nutrient-limited conditions or during transitions to stressful environments. *Mp* uses both EV-mediated and non-EV-mediated pathways to release CARDS toxin, a strategy that allows it to colonize the host, evade the immune system, and promote disease. Both forms of the toxin interact with host cells, with EV association increasing toxin stability, protecting it against proteases, and possibly aiding in toxin transport to distant sites. This combined action of both free and EV-associated toxin forms provides a synergistic advantage for the pathogen. By illustrating the distinct mechanisms of CARDS toxin secretion as a key strategy for *Mp* pathogenesis, our study provides insights into potential EV-based diagnostic approaches and highlights alternative targets for therapeutic interventions aimed at toxin delivery. Understanding how host environmental factors trigger and regulate EV production and CARDS toxin secretion is essential for comprehending the pathogenesis of *Mp* and developing effective therapeutic strategies.

## SUPPLEMENTAL MATERIAL

Supplemental material mbio.02640-25-s0001.docx Additional experimental details.

ASM does not own the copyrights to Supplemental Material that may be linked to, or accessed through, an article. The authors have granted ASM a non-exclusive, world-wide license to publish the Supplemental Material files. Please contact the corresponding author directly for reuse.
