# Supplementary material for: Dual mode of delivery of Mycoplasma pneumoniae CARDS toxin determines the toxin trafficking pathway and cytotoxicity in host cells
Source: mBio. 2025 Nov 28;17(1):e02640-25. doi: 10.1128/mbio.02640-25 (PMC12802180; doi:10.1128/mbio.02640-25)
Supplement: Supplemental material — Additional experimental details. [file mbio.02640-25-s0001.docx]

**Supplemental Materials and Methods**

**Analysis of CARDS toxin secretion in supernatant**

To analyze CARDS toxin secretion, culture supernatants from adherent *Mp* cultures, grown in MM or SP4 broth were collected at specified time intervals (1- 120 h). Cellular debris was removed by centrifugation (12,500 x *g* for 20 min at 4°C) and filtration through a 0.1 μM flow syringe filter (MilliporeSigma). The filtrates were concentrated 100-fold using 10 kDa cut-off Amicon centrifugal filters (MilliporeSigma) and then analyzed for the presence of CARDS toxin by immunoblot. In parallel, the same supernatants were also assessed for the presence of the major adhesin P1 and the cytosolic protein EF-G.

**Purification of extracellular vesicles (EV) and analysis of CARDS toxin**

The mid-log phase *Mp* S1, grown in MM-broth cultures (400 mL each), was used for EV analysis. EV isolation was performed as described before with modifications (1, 2). Briefly, the vesicle-enriched supernatant was concentrated 100-fold and transferred to 4 mL ultracentrifuge tubes. EVs were then isolated by ultracentrifugation (Beckman Coulter, Fisher Scientific, Waltham, MA, USA), at 100,000 × *g* for 90 min at 4°C using a Beckman SW Ti 55 rotor with the brake set to low.

The collected supernatant (EV-free soluble fraction) was concentrated 100-fold and subjected to silver staining to analyze the secreted protein profile, or by antigen capture or immunoblotting for CARDS toxin. The pellet was resuspended in sterile DPBS and subjected to ultracentrifugation as described before. After washing, the pellet was resuspended in 200 μl DPBS and analyzed by transmission electron microscopy (TEM) to confirm the presence of EVs and by silver staining and immunoblotting, to examine the protein profile, and CARDS toxin, respectively.

After confirming the presence of mycoplasma-secreted vesicles and vesicle-associated CARDS toxin in the pellet, further purification of EVs was performed using stepwise density gradient centrifugation. A 10%–45% OptiPrep gradient was prepared in 10 mM HEPES and 0.85% NaCl (2). A 75 µL sample of freshly prepared EVs was mixed with 225 µL of 60% Optiprep to achieve a final concentration of 45% Optiprep. This mixture was then placed in a 4 ml ultracentrifuge tube, and the Optiprep solutions (35%, 30%, 25%, 20%, 15% and 10%) were layered on top in a stepwise manner, and centrifugation was carried out at 180,000 × *g* for 90 min at 4°C with a slow brake using a Beckman SW Ti 55 rotor. After centrifugation, equal volumes of fractions (300 µL each) were collected from the top of the gradient. These fractions were subjected to TEM to visualize the EVs or dot blot to examine CARDS toxin or antigen capture assay to quantify the CARDS toxin concentration.

**TEM analysis of EVs**

Twenty microliters of the collected EV fractions were mounted on Formvar-coated carbon grids and then fixed with 1% glutaraldehyde/4% formaldehyde for 5 minutes at room temperature. The samples were negatively stained using 2.0% (w/v) uranyl acetate (UA), washed three times, and air-dried. The individual grids were examined using a JEOL 1230 transmission electron microscope at an accelerating voltage of 80 kV at the Electron Microscopy Laboratory at the University of Texas Health Science Center at San Antonio.

**CARDS toxin capture assay**

Toxin concentration was measured via a previously reported CARDS toxin capture assay (3). CARDS toxin concentrations in test samples were estimated using a standard curve generated from the dilution series of known amounts of purified rCARDS toxin (10 pg to 10 ng/well) Following a 2-hour incubation and wash with PBST, HRP-conjugated anti-CARDS toxin IgG (0.5 µg/mL) was added and incubated for another 2 h. After a final PBST wash, K-blue TMB substrate was added for 10 minutes. The reaction was stopped with 1N HCl, and the optical density was measured at 450 nm. Buffer-only wells were used as a blank.

**Purification of native free-soluble CARDS toxin**

To purify the secreted CARDS toxin, EV-free soluble supernatants (500-3000 ml) from mycoplasma cultures were collected at 48- or 72-h. The proteins were then precipitated using a stepwise addition of ammonium sulfate (at 20%, 30%, 40%, 50%, 60%, and 70% saturation) (4, 5). The pellets from each fraction were resuspended in 50 mM Tris buffer (pH 8.0) containing protease inhibitors, dialyzed against the same buffer, and then concentrated to 100 µL using a 3-kDa cutoff Amicon centrifugal filter. For further purification, the toxin-containing fractions were adjusted to a concentration of ≥0.2 mg/mL in 50 mM Tris (pH 9). These samples were individually loaded onto a pre-equilibrated Sepharose column at a flow rate of 0.5 mL/min. In each anion exchange purification step, the column was loaded to half its bed volume capacity and washed extensively with the binding buffer (50 mM Tris, pH 8). The toxin was eluted with a Tris-buffered 1 M NaCl solution by stepwise addition of salt concentration. At each salt increment step, the column was eluted with five times the bed volume of buffer, and 5 mL fractions were collected for analysis.

CARDS toxin-containing fractions were pooled and concentrated. For size exclusion chromatography, a 500 µL of the concentrated sample was loaded onto a pre-equilibrated Superose 12 10/300 GL column (Cytiva) and fractionated in 50 mM Tris (pH 8.0), and 100 mM NaCl, at a flow rate of 0.5 mL/min. The resulting fractions were then resolved on a 4-12% NuPAGE gradient gel and silver-stained to analyze the purified protein's homogeneity or transferred to a nitrocellulose membrane for immunoblot analysis.

**Trypsin treatment of CARDS toxin associated with vesicles and in vesicle-free supernatant**

Limited trypsin digestion was performed on purified EVs and vesicle-free supernatant as described previously (6). Trypsin (Promega; 1–10 ng) was incubated with test sample (1–10 µg) at 10:1, 5:1, and 1:1 (ng:µg) ratios for 10 minutes at room temperature. Proteolysis was stopped by boiling the samples in SDS-PAGE sample buffer. After adjusting preparations to load equal amounts of CARDS toxin, samples were analyzed by 10% SDS-PAGE, transferred to a nitrocellulose membrane, and immunoblotted for CARDS toxin.

**References**

1. Gaurivaud P, Ganter S, Villard A, Manso-Silvan L, Chevret D, Boulé C, Monnet V, Tardy F. 2018. Mycoplasmas are no exception to extracellular vesicles release: revisiting old concepts. *PLoS One* 13:e0208160. <https://doi.org/10.1371/journal.pone.0208160>
2. Coelho C, Vij R, Smith DQ, Brady NR, Hamacher-Brady A, Casadevall A. 2020. Study of microbial extracellular vesicles: separation by density gradients, protection assays and labelling for live tracking. *Bio-protocol* 10(2):e3502. <https://doi.org/10.21769/BioProtoc.3502>
3. Kannan TR, Musatovova O, Balasubramanian S, Cagle M, Jordan JL, Krunkosky TM, Davis A, Hardy RD, Baseman JB. 2010. *Mycoplasma pneumoniae* community-acquired respiratory distress syndrome toxin expression reveals growth phase and infection-dependent regulation. *Mol Microbiol* 76:1127–1141. <https://doi.org/10.1111/j.1365-2958.2010.07155.x>
4. Englard S, Seifter S. 1990. Precipitation techniques. Methods Enzymol 182:285–300. <https://doi.org/10.1016/0076-6879(90)82024-V>.
5. Carroll SF, Barbieri JT, Collier RJ. Diphtheria toxin: purification and properties. In: Colowick SP, Kaplan NO, eds. *Methods Enzymol.* 1988;165:68–76. https://doi.org/10.1016/S0076-6879(88)65014-2
6. Kannan TR, Krishnan M, Ramasamy K, Becker A, Pakhomova ON, Hart PJ, Baseman JB. 2014. Functional mapping of community-acquired respiratory distress syndrome (CARDS) toxin of *Mycoplasma pneumoniae* defines regions with ADP-ribosyltransferase, vacuolating, and receptor-binding activities. *Mol Microbiol* 93:568–581.
